# Delving the Role of *Caralluma fimbriata*: An Edible Wild Plant to Mitigate the Biomarkers of Metabolic Syndrome

**DOI:** 10.1155/2022/5720372

**Published:** 2022-06-20

**Authors:** Rimsha Anwar, Roshina Rabail, Allah Rakha, Marcin Bryla, Marek Roszko, Rana Muhammad Aadil, Marek Kieliszek

**Affiliations:** ^1^National Institute of Food Science and Technology, University of Agriculture, Faisalabad 38000, Pakistan; ^2^Department of Food Safety and Chemical Analysis, Prof. Waclaw Dabrowski Institute of Agricultural and Food Biotechnology—State Research Institute, Rakowiecka 36, 02-532 Warsaw, Poland; ^3^Department of Food Biotechnology and Microbiology, Institute of Food Sciences, Warsaw University of Life Sciences—SGGW, Nowoursynowska 159 C, 02-776 Warsaw, Poland

## Abstract

Metabolic syndrome (MS), commonly known as syndrome X or insulin resistance syndrome, is a collection of risk factors for cardiovascular diseases and type II diabetes. MS is believed to impact over a billion individuals worldwide. It is a medical condition defined by visceral obesity, insulin resistance, high blood pressure, and abnormal cholesterol levels, according to the World Health Organization. The current dietary trends are more focused on the use of functional foods and nutraceuticals that are well known for their preventive and curative role against such pathological disorders. *Caralluma fimbriata* is one such medicinal plant that is gaining popularity. It is a wild, edible, succulent roadside shrub with cactus-like leaves. Besides its main nutrient contents, various bioactive constituents have been identified and linked with positive health outcomes of appetite-suppressing, hypolipidemic, antioxidant, hepatoprotective, and anticancer potentials. Hence, such properties make *C. fimbriata* an invaluable plant against MS. The current review compiles recent available literature on *C. fimbriata's* nutritional composition, safety parameters, and therapeutic potential for MS. Summarized data in this review reveals that *C. fimbriata* remains a neglected plant with limited food and therapeutic applications. Yet various studies explored here do prove its positive health-ameliorating outcomes.

## 1. Introduction

Food was mostly acquired from nature or forest resources in nomadic societies [1]. Plants have been the primary source of food, bioactive components, and a necessity for survival and environmental conservation [2]. Likewise, medicinal plants are a real gift from nature to mankind, aiding them in their pursuit of greater health. Such natural foods and their products have been recognized and used as the primary source of therapeutic medications since prehistoric times. They remain a source of effective bioactive compounds that can be utilized directly as medications. According to current estimates, the plant kingdom has about 250,000 species, from which approximately 10% of medicinal plants have been investigated or discovered for the treatment of various diseases [3]. Hence, there is a dire need to explore more hidden components in the plant kingdom.

When compared to conventional medicine, many plants and herb-based therapies have a long history of usage in the prevention and management of various disorders [4]. According to the World Health Organization, the use of herbs, herbal materials, herbal preparations, finished herbal products containing active ingredients of plants, or combinations thereof as drugs are known as herbal, phyto, or botanical medicines [5]. Such medicinal herbs are widely distributed among plant sources and have wide therapeutic applications [6]. The worth of such plants or herbs lies in their secondary metabolites, which are nonnutritive but can exert certain physiological actions in humans against different types of infectious diseases and metabolic disorders [7]. Various plant species throughout the world have been studied for their therapeutic properties, and bioactivity differs from plant to plant, as they pose diverse physiological impacts on the human body [8]. Medicinal plants play an important role in public health, particularly in underdeveloped countries owing to their better affordability and lower toxicity [9]. The extensive use of plants with therapeutic properties does not lead to intoxication, whereas overutilization of allopathic medicines has been associated with adverse effects. Drug residues may lead to the growth of drug-resistant microorganisms that are difficult to treat; hence, the globe is looking for safer alternatives [6].

Recent advances in nutraceuticals and functional foods research have proved that bioactive components in our diet have an important therapeutic role in the treatment of human maladies. Dietary scientists place a high value on isolating nutraceutical bioactive components from food sources [10]. Nutraceuticals, in contrast to traditional diets, are foods or part of food that combine nutritional and pharmacological effects. Hence, nutraceuticals contain any natural component with a nutritional value that has a positive effect on the human body, which are available in the form of powder/pill/dietary supplements or products containing concentrated food derivatives-nutrients. They are typically found in the most common functional foods. Some molecules, micronutrients, and macronutrients including alkaloids, polyphenols, terpenoids, some vitamins (A, B6, B12, C, D, and E), folate, and some trace elements like zinc, iron, selenium, and magnesium are present in these products that are believed to be responsible for the therapeutic effects [11]. Caralluma species are gaining more interest among nutraceutical companies owing to the presence of various phytochemicals with antioxidative potential [12]. Caralluma species have been extensively used for the treatment of various ailments like diabetes, rheumatism, leprosy, paralysis, malaria, and inflammation. A considerable number of active chemicals have been extracted from several Caralluma species. Therefore, scientists are working to produce nutraceuticals from natural products and their derivatives that can aid in enhancing human health while avoiding side effects [13].

Adherence to a nutritious plant-based diet and an active lifestyle, similar to that of our forefathers, may help in the prevention and management of this growing menace of MS. Among plant-based food sources, many indigenous, wild edible plants are still lacking the attention of food scientists, nutritionists, and processors. Thus, there is a need to target such underutilized plant resources to address these lifestyle-related disorders. Unfortunately, still no well-defined studies are conducted to encourage the utilization of historically known wild edible plants as a source of nutrition and therapeutic. Purposely, numerous studies on the nutritional content of wild edible plants are being carried out to raise public attention to their use [2]. One such overlooked plant with significant nutritional and nutraceutical potential is *C. fimbriata*. Henceforth, this review has been designed to compile the latest available scientific literature on *C. fimbriata* to drive the attention of food and nutrition experts, food technologists, dietitians, and clinicians toward its nutritional and nutraceutical utilization at household, industrial, and clinical levels. The primary motivation was to select the underutilized medicinal plant, which is discussed in depth in this review, along with their mode of action and potential mechanism, and could potentially be utilized in the management and prevention of metabolic disorders. This review focuses on different varieties and their important bioactive compounds that have been presented to target MS to open up new avenues for various treatments/therapies [3]. Henceforth, this review has been designed to compile the latest available ten years of scientific studies on *C. fimbriata* to drive the attention of experts in food and nutrition departments towards its encouraged dietary utilization. For this purpose, scientific studies from 2012 onwards were collected using advanced search options on Google Scholar, ScienceDirect, and Scopus with the following keywords: “*Caralluma fimbriata*” OR “*Caralluma adscendens*” OR “Choong” AND “Metabolic Syndrome” OR “Diabetes” OR “Hyperlipidemia” OR “hypertension” OR “Obesity”.

## 2. *C. fimbriata:* An Edible Wild Medicinal Plant

The use of many indigenous medicinal plants is recommended by the WHO owing to their accessibility, affordability, and fewer adverse effects. Britain was the first to cultivate *C. fimbriata* formally in 1830 [14]. Unfortunately, authorities and agricultural entities continue to disregard or overlook the production/cultivation of such wild plants. Furthermore, as a result of overexploitation by the pharmaceutical industry, agriculture, mining, and fodder lopping, these plants are fast disappearing and may become extinct in the near future [15]. Therefore, to meet the global functional food and nutraceutical industry demands, new powerful strategies are required to end this threatening decline [16]. *C. adscendens* var. *fimbriata*, commonly known as *C. fimbriata* [17], is locally known as “Choong” or “Choonga” and “Kalli moolian” or” Karallamu” in Pakistan and India, respectively [18]. It is a resilient roadside shrub with cactus-like leaves and is well known in Ayurvedic medicine. The plant is about 20-30 cm tall, erect, branching herb with leafless four-angled green stem tapered to the tips. Its leaves are small that only appear on young branches and fall off quickly leaving spiky projections. Flowers bloom either singly or in groups at the ends of branches on short stalks. Its flowers are 2 cm in diameter, with small petals of purple color marked with golden and hairy borders [19]. Natural bioactive supplements are becoming increasingly popular for weight reduction, and *C. fimbriata* is currently regarded as one such functional plant that has exhibited potential outcomes [20].

*C. fimbriata* is an edible wild medicinal plant that grows in dry areas and is known as “famine food” by tribal Indians. Traditionally, it is eaten as a pickle or vegetable [21]. Through appropriate selection and climate adjustments, this wild plant can be easily adapted for its large-scale cultivation [16]. History has reported its use during long hunting periods since hunting tribes in the form of chewable portions or chunks of the *C. fimbriata* to suppress hunger and quench thirst. No incidence of adverse effects has been reported after the use of *C. fimbriata* in the Indian subcontinent [22]. It contains pregnane glycosides that are known to suppress hunger and increase endurance. The plant has been studied for its antihyperglycemic and hypolipidemic properties, as well as hepatoprotective and antioxidant activities, yielding significant results [18, 23]. It is also used to treat pain, fever, and inflammation. The plant is commonly consumed by ethnic populations of Central India to manage obesity [24]. It also stimulates the central nervous system, and its therapeutic benefits are attributable to the pregnane group of glycosides found abundantly in them [25]. Flavone glycosides, pregnane glycosides, saponins, triterpenoids, and other flavonoids are important phytochemical constituents of *C. fimbriata* that have been studied against various pathological conditions and metabolic disorders. Pregnane glycosides are the secondary metabolites of *C. fimbriata* that are the steroidal compounds conjugated with sugar moiety [26]. They are related to altered lipid metabolism and inhibit the synthesis of fatty acids [18, 27]. They also act on the hypothalamus and cortisol, causing a feeling of fullness, thereby reducing hunger, and are most likely responsible for appetite suppression [28]. This occurs without any side effects compared to the known appetite suppressant drugs [29].

*C. fimbriata* extract (CFE) is commercially available in several countries including Australia and New Zealand [30]. CFE has been granted generally recognized as safe (GRAS) classification for use as a nutraceutical in the fight against the world's most significant public health problem (i.e., obesity) [31]. For example, GenaSlim is a brand of CFE that has been approved for weight loss programs. The use of CFE as a therapeutic intervention is well known in Ayurvedic medicine [32]. CFE can also be used as a natural antioxidant [33]. Other therapeutic applications of CFE reported in the literature include carminative, febrifugal, anthelmintic, antirheumatic, anti-inflammatory, antinociceptive, and antioxidant actions [25]. Therefore, CFE could serve as an appropriate chemically tested, safe, and effective appetite suppressant resulting in weight loss, blood glucose, and lipid reduction [34]. In addition, it has been used against malaria, hyperglycemia, ulcers, cancers, and other diseases. Future research for antiobesity and appetite-suppressant medications and nutraceuticals should focus on this significant phytochemical-pregnane glycoside [31]. It has been known to have hypolipidemic, antioxidant, hepatoprotective, antiobesogenic, and anticancer properties with few side effects [23].

**Figure 1:**
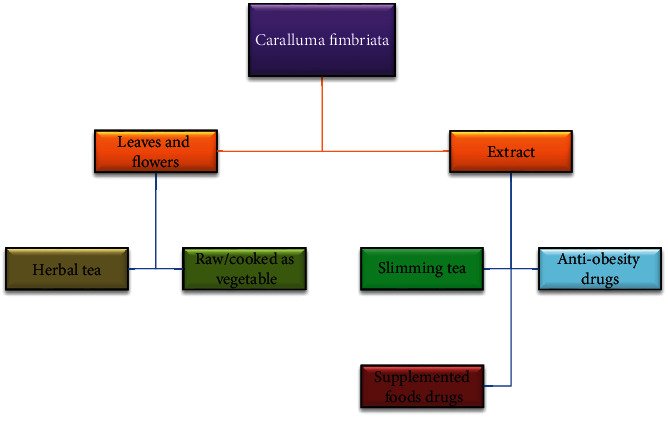
*C. fimbriata* utilization in various dietary and supplemental forms.

In the Indian Health Ministry's comprehensive compilation of medicinal plants, it is listed as a vegetable [22]. Also, it is classified as famine food, hunger suppressor, and thirst quencher in Indian Materia Medica [35]. Its aerial parts are traditionally used as a culinary herb and cooked with meat during the winter [24]. For decades, tribal communities in India have consumed this as a traditional vegetable alternative. CFE is also largely available and easy to consume despite its bitter taste. Its safety and toxicity profile has been thoroughly investigated [30]. While in the semiarid areas of Pakistan, its species have been used as emergency food for centuries [21]. *C. fimbriata's* dietary or supplemental utilization is shown in Figure 1.

## 3. Nutritional and Phytochemical Constituents of *C. fimbriata*

*C. fimbriata* has about 45% moisture, 9% ash, 4.8% fat, 0.67% fiber, 0.66% protein, 40% carbohydrates, and 207 kcal/100 grams in terms of its nutritive value with a significant load of iron, manganese, zinc, and copper [36]. Another study indicated some variations but significant amounts of fats, ash, sugars, and caloric contents accounting for 8%, 6%, 30%, and 554 kcal/100 grams, respectively [20]. The examination of amino acid composition (mg/100 g dry weight) of aerial parts of *C. fimbriata* revealed aspartic acid 21.6, glutamic acid negligible, alanine 120.72, methionine 22.56, tyrosine 130.08, lysine 316.56, threonine negligible, proline 483.8, isoleucine 1578.24, phenylalanine 141.58, tryptophan 157.36, glycine 108.29, arginine 51.58, histidine 84.48, and valine 342.95. Likewise, moisture 82%, lipid 5.6%, carbohydrates 55.4%, protein 3.5%, total free amino acid 27.5%, crude fiber 15.3%, and ash 2.1% were also found [27].  Table 1 elaborates on the phytochemicals identified in *C. fimbriata* along with their bioactive potentials reported in various studies. Future investigations for bioactive peptides and genetic variations are still awaited in this regard [37, 38].

Extraction of CFE based on various extraction methods along with the detection and identification of bioactive nonnutritive compounds of *C. fimbriata* as explained in Table 2 revealed considerable amounts of steroids, coumarin, phytosterol, flavonoids, and alkaloids and medium levels of diterpenes and saponins while the relative absence of anthocyanins, phenols, tannins, phlobatannins, and cardinal glycosides. Similarly, steroids, coumarin, proteins, carbohydrates, diterpenes, phytosterol, flavonoids, saponins, and alkaloids were identified positively along with the high concentration of minerals and elemental compounds [22]. One more study looked into the existence of phytochemical ingredients, total phenolics, and flavonoid contents along with the antioxidant capacity of whole plant CFE utilizing various in vitro assays based on Soxhlet's extraction, 2,2-diphenyl-1-picrylhydrazyl (DPPH), nitric oxide (NO) scavenging, and ferric reducing antioxidant power test (FRAP) techniques. The total phenolic and flavonoid content of the plant ethanolic extract were found to be 80.08 ± 0.629 mg and 70.88 ± 1.170 mg, respectively. The ethanolic extract has higher antioxidant activity than the other extracts as measured by DPPH, NO, and FRAP [8]. Moreover, a study was conducted to check the phytochemical constituents of CFE using different solvents (aqueous, ethanolic, methanolic, and ethyl acetate). Methanolic extract revealed that the whole plant was rich in alkaloids, flavonoids, glycosides, phenolic compounds, saponins, and quinones. Mainly oleic acid (21.08%) and n-hexadecanoic acid (44.23%) were detected [61]. Another study indicated that *C. fimbriata* had total free phenolic 13.56 mg/100 g, while antinutritional compounds, namely, tannins (112 mg/100 g) and oxalates (125 mg/100 g), were also observed [2]. CFE included roughly 12% pregnane glycosides and 1.3% polyphenols according to phytochemical analyses. The current findings point to presumed *C. fimbriata* effects on ingestive behavior which are most likely mediated by cerebral and peripheral processes [55].

## 4. Therapeutic Potential and Bioactive Compounds of Genus *Caralluma*

There are almost 18,000 flowering plant species that account for nearly 11% of all plants in the world [6]. Caralluma is among such beneficial medicinal flowering plants that belong to the family Apocynaceae and is found throughout Asia (Afghanistan, India, Iran, Pakistan, and Sri Lanka), Africa, Arabian Peninsula, Canary Islands, and Southeast Europe. The name “Caralluma” is derived from the Arabic word “qarh al-luhum” meaning “flesh wound” or “abscess” [24]. Caralluma has approximately 200 genera and 2500 species distributed all over the world, especially in tropical Asia and the Mediterranean regions [31, 62]. The chemical composition of different species of Caralluma depends on the growing conditions, morphological features, and genetic characterization. The presence of different phytochemical constituents makes them unique and diversified from one another [63]. Different species of Caralluma, i.e., *C. stalagmifera*, *C. adscendens* var. *attenuate*, *C. tuberculata*, *C. arabica*, *C. attenuate* Wight, *C. burchardii*, *C. edulis*, *C. europaea*, *C. flava*, *C. indica*, *C. lasiantha*, *C. negevensis*, *C. sinaica*, and *C. umbellata* Haw, have been reported in the literature for their beneficial outcomes. These species have antimalarial, antiulcer, antioxidant, and antiproliferative properties and are extensively used in traditional medicines for the treatment of diabetes, rheumatism, paralysis, leprosy, and inflammation.  Table 3 elaborates some of the major bioactive components of the Caralluma genus that are known for their role against MS including pregnane glycosides, flavonoid glycosides, flavones, magastigmane glycosides, pregnane steroids, steroidal glycosides, saturated and unsaturated hydrocarbons, aromatic and nonaromatic volatile chemicals, and *β*-sitosterol [31].

## 5. Metabolic Syndrome (MS)

Recent advancements in contemporary civilization have resulted in a convenient lifestyle accompanied by poor nutrition, fast food intake, lack of physical activity, and excessive stress which are the major causes of many metabolic disorders. Among these, diabetes, hypertension, and hyperlipidemia are the most common ones that can lead to serious ailments, i.e., cardiovascular diseases, atherosclerosis, cerebrovascular diseases, and cancers. All such pathological conditions have led to increased mortality attributable to noncommunicable diseases related. Metabolic syndrome (MS) is a collection of interrelated conditions that may lead to type 2 diabetes and cardiovascular diseases. Dyslipidemia (high levels of low-density lipoprotein (LDL), triglycerides (TG), or low levels of high-density lipoprotein (HDL)), high blood pressure, obesity, poor glucose metabolism, and/or insulin resistance are the key biomarkers of MS [77]. MS, also known as syndrome X or insulin resistance syndrome, is a group of risk factors for heart diseases affecting over a billion people worldwide [78]. MS is the leading cause of sickness and mortality in both developed and developing countries with significant economic consequences [79]. Obesity, diabetes mellitus, nonalcoholic fatty liver disease, stroke, and cardiovascular diseases have all reached epidemic proportions in recent decades and have become crucial for clinical and translational research [80]. Interestingly, each of these disorders has distinct physiological and clinical symptoms; they appear to share pathological traits such as intracellular stress and inflammation caused by metabolic disturbances resulting from excessive nutrition which is frequently aggravated by a modern sedentary lifestyle [81].

MS, whether inherited or acquired, has been regarded as a critically important health issue worldwide that needs quick and effective management. A significant proportion of these ailments is still difficult to manage effectively despite enormous advancements in contemporary medicines. Multiple underlying causes including hereditary transmission, imbalanced diet, and unhealthy lifestyle are attributed to the growing burden of the said diseases [4]. Common biomarkers used for early detection and differential diagnosis of numerous metabolic disorders have been called into question owing to significant interindividual variability. For example, not all obese individuals have metabolic issues later in life, and 25–40% of them can lead healthy lives as well. Hence, early screening of high-risk individuals is critical for the prevention and management of MS [80]. The most prevalent features of the pathogenesis of MS are insulin resistance and visceral adiposity, even though it is diagnosed based on at least three metabolic abnormalities. Currently, scientists are concentrating their efforts on dietary components that have the potential to prevent a variety of chronic issues. As a result, consumers are shifting away from dietary supplements toward more nutritious eating habits [77].

## 6. Therapeutical Potential of *C. fimbriata* against MS

Various scientific studies have established the baselines for the potent therapeutical potential of *C. fimbriata* due to the wide range of phytochemicals therein with scientifically proven nutraceutical active potentials (as elaborated in Tables 1 and 2). Many of those nutraceutical components have been found protective against ailments of MS, i.e., diabetes mellitus, hypertension, hyperlipidemia, and obesity, for whom mechanisms of action have been listed in Table 4. Studies investigating the therapeutical potential of *C. fimbriata* against MS have been enlisted in Table 5, and the important description of these studies has been discussed here under the relevant subheadings of this section.

### 6.1. Antidiabetic Potential of *C. fimbriata*

Diabetes mellitus is a metabolic disorder characterized by high blood glucose levels caused by insufficient or ineffective pancreatic insulin. According to WHO, diabetes affects 3% of the world's population, and the prevalence is anticipated to double to 6.3% by 2025 [79]. According to statistics approximately 79.4 million people in India alone will be infected with the disease by 2030 [23]. As the prevalence of diseases rises, experts must look for more effective therapies with fewer side effects. Diabetes is treated with a wide variety of pharmacological medications. Although there are various types of oral hypoglycemic drugs and insulins available for the treatment of diabetes mellitus, people are increasingly seeking natural antidiabetic therapies with fewer adverse effects [86]. Currently, existing allopathic drugs have the potential to produce obesity and hyperandrogenemia while reducing blood glucose levels. Traditional medicinal herbs are used to treat diabetes mellitus all around the globe since they are less toxic and less costly and have fewer adverse effects than synthetic medicines. As a result, research on drugs derived from traditional medicinal plants has grown in importance [87].

Diet is one of the key elements that impact metabolic homeostasis including impaired glucose and lipid metabolism [18]. Insulin resistance and related disorders such as obesity and type 2 diabetes mellitus are linked to a high dietary fat intake. Controlling postprandial hyperglycemia and fat absorption through inhibition of enzymes responsible for glycoside and lipid hydrolysis should help to reduce MS complications [26]. The hypoglycemic action of the pregnane glycoside is mainly attributed to the lowering of intestinal glucose absorption or stimulation of pancreatic insulin production [58].

**Figure 2:**
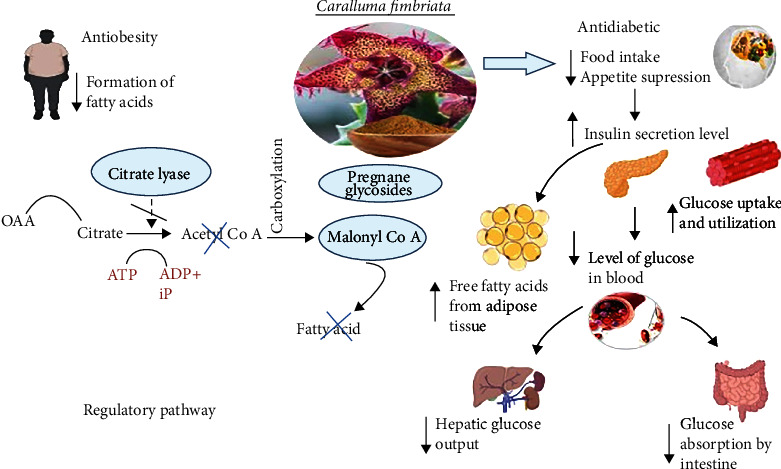
Regulatory pathways in the presence of *C. fimbriata* represent antiobesity and antidiabetic potential. ^∗^OAA: oxaloacetate; acetyl-CoA: acetyl coenzyme A; ATP: adenosine triphosphate; ADP: adenosine diphosphate; iP: inorganic phosphate^∗^; ↓: decreasing/downregulation; ↑: increasing/upregulation.

Figure 2 represents the regulatory mechanism involved in the antidiabetic and antiobesity potentials of *C. fimbriata.* A study was carried out to assess the CFE for its modulatory effects on carbohydrate metabolism and inhibition of *α*-amylase as measured by key enzyme activities and changes in glycogen content (liver and muscle) in a high-fat diet- (HFD-) fed diabetic rats. The results revealed that both CFE and metformin administration prevented changes in the activities of glucose metabolism enzymes and dramatically restored glycogen levels in the liver and muscle of HFD-fed rats. Furthermore, muscle myofiber degeneration and fat accumulation were less pronounced in these groups. Such findings suggest that CFE is beneficial in regulating carbohydrate metabolism associated with high-calorie diet consumption [18]. Another study investigated the antihyperglycemic properties of CFE. Purposely, *α*-amylase and *α*-glucosidase inhibitory assays were performed on different concentrations (1–1000 *μ*g/mL) of the *C. fimbriata* leaf extract with controlled acarbose. The extract was used (100 *μ*g/mL) in a glucose uptake experiment along with metformin and insulin as control treatments. CFE displayed a significant inhibitory effect on glucose metabolism enzymes. CFE showed highest glucose absorption (66.32 ± 0.29%) at 100 *μ*g/mL, 74.44 ± 1.72% for metformin (10 g/mL), and 85.55 ± 1.14% for insulin (10 *μ*mM). The results confirmed CFE to be safe because the IC_50_ of extract and metformin in the cell line examined was 1000 *μ*g/mL and 1000 *μ*M, respectively [23].

Another investigation was aimed at checking the effects of CFE on insulin resistance and oxidative stress caused by the HFD in Wistar rats. The rats were given the HFD and CFE (200 mg/kg body weight/day) for 90 days. Hyperglycemia, hyperinsulinemia, hyperleptinemia, hypertriglyceridemia, and reduced insulin sensitivity were developed as a result of HFD. The rats fed on HFD had increased levels of lipid peroxidation, protein oxidation, lower growth-stimulating hormone (GSH) levels, and reduced enzymatic antioxidant activity in the liver, whereas CFE therapy corrected all these abnormalities. Moreover, the study revealed that CFE helped to reduce insulin resistance and oxidative stress caused by HFD [14]. Another study was done to check the effect of CFE in diabetic rats. At different treatment periods, oral administration of CFE to diabetic rats at doses of 100 and 200 mg/kg body weight resulted in a significant reduction in blood glucose. Other parameters of the study included body weight, glycosylated hemoglobin (HbA1c), plasma insulin, total protein, liver, and renal biomarkers. The CFE-treated diabetic rats considerably recovered from hepatotoxicity, diabetes, and renal toxicity [87].

### 6.2. Antihypertensive Potential of *C. fimbriata*

Hypertension is defined as systolic blood pressure (≥140 mmHg) and diastolic blood pressure (≥90 mmHg). The use of antihypertensive medication is a major public health concern around the world. In 2000, 26.4% of people in the world had hypertension, which was expected to rise to 29.2% by 2025. In 2017, high blood pressure caused 10.4 million deaths along with 218 million disability-adjusted life-span worldwide [96]. Hypertension is among the major risk factors for heart diseases and strokes [97].

The development of atherosclerotic lesions due to the alterations in endothelial cell functioning contributes to the progression of cardiovascular disease. Increased generation of reactive oxygen species, oxidative stress, and reduced bioavailability of nitric oxide are all related to endothelial dysfunction, which can lead to arterial hardening and an increase in blood pressure. Flavonoids are the secondary metabolites and important constituents of Caralluma that act as antihypertensive agents by restoring endothelial function and by affecting the levels of nitric oxide [84]. The administration of CFE positively affected the metabolic markers in HFD-fed Wistar rats. After the intervention, organ weights, belly circumference, total cholesterol, triglycerides, and liver lipid content were all measured. The CFE appeared to have potential appetite-suppressing, antiobesity, and antihypertensive outcomes [92].

### 6.3. Antiobesity Potential of *C. fimbriata*

Obesity is one of the most serious public health problems of the 21^st^ century. Obesity and overweight are both defined as an abnormal or excessive buildup of fat mass that has the potential to harm human health. According to the WHO, the global prevalence of obesity nearly quadrupled between 1975 and 2016 [98]. In 2016, the World Health Organization estimated that 39% of adults (1.9 billion) and 13% (650 million) were overweight or obese, respectively [99]. Obesity has been linked to a variety of ailments including heart disease, cancer, arthritis, hypertension, stroke, hyperlipidemia, and diabetes [100]. According to the National Health and Nutrition Examination Survey, the percentage of adults over the age of 20 who are obese in the United States grew from 13% to 30% between 1960 and 2000 [14]. Obesity treatment usually includes dietary modulations and physical activity adaptations in the form of lifestyle changes. When lifestyle modifications are ineffective, pharmacotherapy is generally prescribed as a second-line treatment [92]. *C. fimbriata* aids in fat burning and hunger suppression. It works by inhibiting the citrate lyase enzyme, causing our bodies to stop producing fat and also inhibits the production of malonyl coenzyme A, which promotes the oxidation of stored fatty acids. Hence, it can be an effective intervention to block fat synthesis. Furthermore, it helps in the burning of stored fat resulting in weight loss [85].

A recent study was carried out to investigate how CFE affects satiety indicators and body composition in overweight adults. For this purpose, 83 men and women aged 20 to 50 years old took 1 g/day of CFE for 16 weeks. The placebo group's plasma leptin levels rose at week 16, whereas the CFE group remained the same. In addition, the CFE group's waist circumference decreased by 2.7 cm. The placebo group gained weight, but the CFE group dropped weight from the start (0.37 kg gain versus 0.33 kg loss). Evidently, the CFE has been shown to help people maintain their weight [20].

The CFE has also been used as a “natural slimming” dietary supplement because of its high concentration of pregnane glycosides. An efficacy trial on “Slimaluma” containing 100 mg/kg of CFE in female rats effectively modulated ingestive behavior and regulation of the brain neuropeptide Y (NPY) and orexin (ORX). Interference of CFE with the enzymes amylase and lipase has been examined in vitro as a possible adverse effect mechanism. The chemical composition of CFE was also figured out using NMR and spectrophotometric studies. According to the results of in vivo experiment, CFE did not affect blood parameters and liver/gut histomorphology. Increased water consumption and hypothalamic levels of NPY and ORX peptides were shown to minimize body weight gain [55]. A study was conducted to evaluate the efficacy of CFE in overweight and obese individuals. For this purpose, 89 patients were randomized into a treatment group and a placebo group. A capsule of 500 mg of CFE was given for 12 weeks daily. The results revealed that there were no significant changes in weight, body mass index, waist, and hip circumference [91]. Another study was conducted to investigate the effect of CFE on body weight, appetite, and lipid profile in obese rats. For this purpose, CFE was given 100 mg/kg for 50 days. The findings showed a significant reduction in body weight and lipid profile [93]. Likewise, the effect of CFE (500 mg twice daily) with restricted dietary intake and physical exercise was investigated in 33 overweight and obese subjects. The final results displayed a significant decline in BMI, body weight, hip circumference, total fat, and systolic blood pressure [95].

### 6.4. Antihyperlipidemic Potential of *C. fimbriata*

A study investigated the hypolipidemic potential of *C. fimbriata*. The results of the study revealed that HFD-induced heart damage in rats had increased the serum lipid profile such as total lipids, triglycerides, total cholesterol, and free fatty acids. All such abnormally raised values were considerably mitigated by CFE therapy due to an increase in lipid peroxidation and protein oxidation. The activity of antioxidant enzymes, i.e., creatine kinase, aldose reductase, and sorbitol dehydrogenase, was also significantly reduced. The study indicated that CFE was an adjuvant medication to prevent or manage heart damage caused by the HFD [62]. Similarly, a study compared the protective effects of CFE and metformin against HFD-mediated oxidative stress, which contributes to pancreatic fibrosis in Wistar rats. Reduced glutathione, lipid oxidation, protein oxidation, and activity of antioxidant and polyol pathway enzymes, aldose reductase, and sorbitol dehydrogenase were measured in the pancreas after 90 days of intervention. Both CFE and metformin groups were able to avoid oxidative damage, while CFE had a better antioxidant status. The CFE therapy reduced acinar cell degeneration, necrosis, edema, and bleeding. Moreover, CFE proved an adjuvant therapy in the prevention or managing pancreatic damage caused by HFD [89]. Another study was conducted to check the effect of CFE (200 mg/kg body weight) and metformin (20 mg/kg body weight) on HFD-induced changes in lipid metabolism of Wister rats. Hypercholesterolemia and hypertriglyceridemia with decreased HDL cholesterol, LDL cholesterol, and VLDL cholesterol were observed in HFD-fed group, while CFE/metformin proved valuable in ameliorating lipid metabolism biomarkers [35]. Similarly, a study was carried out to evaluate the impact of CFE (100 mg/kg/day) on lipid profile and body weight for the duration of 50 days. The results showed a significant reduction in lipid profile and body weight gain [94].

## 7. Nutraceutical Potentials of *C. fimbriata* against Other Diseases

### 7.1. Appetite-Suppressant Potential of *C. fimbriata*

Five mechanisms by which herbal medicines and their products can help people lose weight are as follows: (1) appetite suppression, thereby reducing energy intake, (2) stimulation of thermogenesis, (3) inhibition of pancreatic lipase activity, (4) reducing fat absorption, and (5) alteration in lipogenesis [99] (elaborated in Figure 1). Caralluma is a natural appetite suppressant that can be used as a weight loss supplement. Supplementation with *C. fimbriata* can lead to a clinically meaningful reduction in central adiposity, a key component of MS associated with other risk factors such as elevated blood pressure and cardiovascular disease. It contains pregnane glycosides, a class of naturally occurring compounds believed to inhibit the formation of fat [19]. A study revealed that a 14-year-old girl with Prader-Willi syndrome (PWS) was successfully treated for hyperphagia. At the age of two, the child began taking a supplemented CFE for appetite control. The CFE was given as a drink once a day, and the dose was gradually increased for appetite suppression. After extensive testing, blood count, vitamins, essential minerals, HbA1c, IGF-1, liver, and thyroid function tests were all within normal ranges. The study indicated that CFE may be effective in preventing hyperphagia and obesity in PWS through early intervention [32]. Another study explicated that supplementation of CFE reduced hyperphagia in children and adolescents with PWS. The CFE supplementation resulted in a substantial drop in hyperphagia at the highest dose of 1000 mg/day. The findings suggest that CFE may have a positive impact on PWS management [30].

### 7.2. Anticarcinogenic Potential of *C. fimbriata*

The Indian plant *C. fimbriata* has been shown to have cytotoxic activities against human colon cancer cell lines [88]. Human colon cancer is a malignant tumor of the digestive tract that is one of the major causes of death in both men and women worldwide. Colon cancer is currently the third most common cancer type in humans, the fourth most common cause of cancer-related death, and the second most common cancer type in terms of people living with cancer about 5 years after diagnosis. Every year, around 694,000 people worldwide die from colon cancer [88]. A study revealed against the KB mouth cell line CFE showed good antiproliferative action after assessing the drug's practical application and clinical efficacy; it may be utilized to treat oral cancer [92]. A study was done to see if CFE affected the COLO 320 cell line's cytotoxicity. For 24 hours, COLO 320 cells were treated with varying amounts of CFE (100–300 g/mL). In COLO 320 human colon cancer cells, CFE increased cytotoxicity. The most cytotoxic effect was seen at the highest dose of 300 *μ*g with an IC_50_ of 233.87 *μ*g. The results revealed that inducing cytotoxicity in COLO 320 cells with CFE reduced cell growth [88]. The 2,5-diphenyl2H-tetrazolium bromide (MTT) cell viability experiment was done on KB cell lines that had been treated for 24 hours with increasing doses of ethanolic extract of *C. fimbriata*. The result was analyzed using cyclophosphamide as a positive control. In another study, viability of the cells at maximum concentration was found to be 28.47%, while the control showed 21.87% cell viability. CFE showed dose-dependent cytotoxicity with maximum toxicity of 71.52% at the maximum concentration. The inhibitory concentration (IC_50_) value of CFE was found to be 28.39 *μ*g/mL. Against the KB mouth cell line, CFE showed good antiproliferative action [88].

### 7.3. Antidepressant and Anti-Antioxidative Potential of *C. fimbriata*

In Western cultures, the global prevalence of diagnosed anxiety disorders is greater than 10%. There are also a significant number of people who suffer from anxiety but have not been diagnosed with a mental illness. Stress and subclinical (mild to moderate) anxiety are now widely accepted as lowering the quality of life [28]. Increased formation of free radicals causes oxidative stress which is linked to lower antioxidant levels in the myocardium and plays a crucial role in cardiovascular disease [62]. A study was done to check the effect of CFE in lowering anxiety and stress in healthy individuals. An 8-week double-blind randomized clinical experiment in which 97 people with mild to moderate anxiety were administered either 500 mg CFE (n =49) or 500 mg placebo (n =48) in a double-blind placebo-controlled trial. The GAD-7, perceived stress scale (PSS), positive and negative affect schedule (PANAS), and salivary cortisol were used to assess the timing of treatment impact at baseline, week 4, and week 8. CFE proved superior to placebo in lowering subclinical anxiety and stress over 8 weeks [28]. *C. fimbriata* revealed protection of testes of male Wister rats from oxidative stress caused by the HFD. CFE was given orally to rats in groups C+CFE and HFD+CFE for 90 days (200 mg/kg body weight). HFD-fed rats had greater levels of lipid peroxidation, protein oxidation, polyol pathway enzymes, reduced GSH levels, and decreased antioxidant activity in their testes, but CFE therapy corrected all of these abnormalities. CFE provided considerable protection against HFD-induced testicular oxidative damage [39]. A study used *C. fimbriata* as a natural antioxidant succulent cactus. The results revealed that fresh CFE had the maximum phenolic level of 96.4 ± 0.1 mg GAE/g, while the raw potato extract had a much lower phenolic content of 27.4 ± 0.3 mg GAE/g. The raw potato had 38.8 ± 0.2 mg QE/g of flavonoid content, while the fresh CFE had 54.4 ± 0.1 mg QE/g. After immersion treatment, CFE was found more effective in reducing acrylamide levels in French fries (42.5 g/kg) [33].

## 8. Safety and Tolerability/Toxicity Assessment

For human consumption, *C. fimbriata* is considered pharmacologically safe due to its natural occurrence and less toxicity. However, in some cases, it has been reported with no serious adverse effects by subjects of the study. The reported side effects were minor and limited to mild gastrointestinal symptoms such as constipation, flatulence, abdominal distention, and gastritis. All the above symptoms disappeared within a week, and the drug was shown to be nontoxic up to a dose of 2000 mg/kg. Hence, standardized extract of *C. fimbriata* was clinically tested and proven with no known side effects and was approved by TGA (Therapeutic Good Administration, Australia) [101]. Similarly, a study was done to check the toxicological assessment of CFE at different doses of 100, 300, and 1000 mg/kg body weight for six months in Sprague Dawley rats. No treatment-related toxicity or deaths were seen up to the maximum dose [102]. Another study was conducted to check the limitation of CFE, resulted in no reported adverse effects at the recommended dose of 1000 mg/kg [30]. Moreover, an efficacy study revealed that it was found to be nontoxic even up to the dose of 2000 mg/kg body weight [103].

## 9. Conclusion

The Caralluma genus comprises 260 species, and almost all of them have been considerably used for the treatment of various diseases. A large number of bioactive compounds like pregnane glycosides, megastigmane glycosides, alkaloids, quercetin, and flavone glycosides have been isolated from Caralluma species and used against obesity, diabetes, hypertension, ulcers, and cancer. One of these species, *C. fimbriata*, is an indigenous, wild, edible, succulent roadside shrub with cactus-like leaves. Exploration of the nutritional and nutraceutical potential of *C. fimbriata* has revealed significant bioactive constituents that have shown amelioration in cardiometabolic biomarkers, hyperglycemia, obesity, and appetite control. Hence, this neglected and underutilized vegetable should be more cultivated for its regular dietary utilization. The summarized data of this review has revealed that there is still very little work done on *C. fimbriata*. Therefore, more research on such a hidden miraculous plant and its reported active biomolecules should be done to authenticate its GRAS status. Further phytochemical and pharmacological research with more work done on innovative ideas to incorporate CFE in diet or supplements should be done to address critical health concerns prevailing in developed as well as developing countries. As this plant still needs to get spotlighted in food and biomedical science, therefore, future investigations are welcomed to identify its therapeutic potential against different diseases either metabolic syndrome or not. Such studies can serve as a scientific baseline for designing a safer nutraceutical approach to these diseases.

## Figures and Tables

**Table 1:** Phytochemical constituents in *C. fimbriata.*

Phytochemical constituents	Bioactive potential	Reference
Total polyphenolic compounds	Antioxidant; cardioprotective; neuroprotective; and antihyperglycemic	[8, 22, 39, 40, 41, 42]
Flavonoids	Antioxidant; antiaging; anti-inflammatory; antifungal; immunomodulatory; cardioprotective; antiviral; antimicrobian; antibacterial; and antiparasitic	[8, 22, 34, 40, 43]
Saponins	Antitumor; antioxidative; anti-inflammatory; antidiabetic; and neuroprotective	[8, 22, 35, 40, 44]
Alkaloids	Antiadipogenic; antihyperglycemic; and antioxidant	[8, 22, 39, 42, 45]
Anthocyanins	Protective against cardiovascular diseases; cancers; neurodegenerative disorders; and aging-associated bone loss	[34, 46, 47]
Coumarins	Antioxidants; antitumor	[8, 22, 35, 48]
Tannins/gallic-tannins	Antiulcerative; anti-inflammatory; antioxidant; antidiabetic; anticancer; and cardioprotective	[8, 39, 40, 49, 50]
Steroids	—	[22, 34].
Diterpenes	Antiobesogenic; antihyperlipidemic; and anticarcinogenic	[51, 52, 22]
Phytosterol	Antihyperlipidemic; anticancer; antiapoptotic; cardioprotective; and anti-inflammatory	[8, 40, 53]
Quinones	—	[8]
Terpenoids	Anti-inflammatory; antitumor; and antiparasitic	[8]
Anthraquinones	Diuretic; antibacterial; antiulcer; anti-inflammatory; anticancer; and antinociceptive	[8, 54]
Pregnane glycosides	Antidiabetic; antiobesity; antinociceptive; antiulcer; anti-inflammatory; antiarthritis; and wound healing activities	[31, 55–58]
Pregnane steroids	—	[31]
Trigonelline	Anti-inflammatory; antioxidant; antipathogenic; and antiaging	[55, 59]
Glycosides	—	[60]

**Table 2:** Extraction and biochemical activity of *C. fimbriata* extracts (CFE).

Extraction	Methodology	Result	References
Shade dried, powdered, and extracted in Soxhlet's apparatus using several solvents including pet ether (PE), chloroform (C), ethyl acetate (EA), ethanol (E), and aqueous (A)	Phytochemical screening: TPC, TFC, and antioxidant radical scavenging activity analysis	Ethanolic extraction has shown a better antioxidant profile, whereas saponins were found in extracts of PE, C, and E Alkaloids and phenols in EA and E Anthraquinones in C, EA, and E Carbohydrates in C Flavonoids in E Steroids in C and EA Coumarins in EA, E, and A Quinones in C, EA, and E Tannins in C and EA	[8]
Dried (fluidized-bed drier at 45°C for 60 min) Homogenized (food processor) Mixed with hot water (at 65°C for 3 h in a temperature-controlled incubator shaker) Cooled (room temperature) Filtered (No.1 sinter glass funnel) Centrifuged extract	Phenolic and flavonoid content were analyzed	*C. fimbriata* acted as a natural antioxidant against acrylamide and oxidative deterioration due to higher phenolics and l flavonoid content	[33]
Slimaluma®, a dry ethanolic extract from the aerial portions of *C. fimbriata*	Phytochemical analysis using NMR spectroscopy	Amino acids: leucine, isoleucine, alanine, glutamine, and tryptophan Organic acids: lactate, acetate, and formate Carbohydrates: glucose, sucrose trigonelline, and pregnanes	[55]
Shade drying method	Nutritional and phytochemical analysis	Following phytochemicals detected steroid, coumarin, proteins, carbohydrates, diterpenes, phytosterol, flavonoids, and alkaloids	[22]
Shade drying method	Free phenolics and antinutritional content were analyzed	Total free phenolics and antinutritional content (tannin and oxalates) were detected positively	[2]
Shade dried, powdered, and extracted in Soxhlet's apparatus using several solvents including chloroform, ethyl acetate, methanol, and water	Phenolic and flavonoid content were analyzed	Methanol and water extraction has shown better antioxidant profiles and phenolic compounds	[21]
Shade drying method	The amino acid composition was checked	Following phytochemicals detected aspartic acid, alanine, methionine, tyrosine, lysine, isoleucine, glycine, and phenylalanine, negligible amounts detected for glutamic acid and threonine	[27]
Shade drying method using several extracts Aqueous, ethyl acetate, ethanolic, and methanolic extracts	GC-MS analyses were done	Alkaloids, flavonoids, glycosides, phenolic compounds, saponins, quinones, oleic acid, and n-hexadecanoic acid were positively detected in methanolic extract	[61]

**Table 3:** Bioactive compounds isolated from various *Caralluma* species.

Bioactive component	Extraction	Therapeutic potential	Species variety	Reference
Pregnane glycosides (27 compounds)	Methanolic extraction	High cytotoxic activities	*C. gracilis* *C. dalzielii*	[64]
Novel Pregnane glycoside	Ether eluates of methanol and benzene fractions of ethanolic extract	—	*C. umbellate*	[64]
New pregnane glycosides (2-13)	Ethanolic and butanolic extraction	Appetite suppressant Antiobesity High cytotoxic activity	*C. fimbriata*	[64]
New pregnane glycosides (1-20)	Chloroform and methanol extracts	—	*C. negevensis* *C. russeliana* *C. sinaica*	[64]
Four tetrasaccharide pregnane glycosides (desflavasides A-D)	Sap	Antidiabetic and antiulcer	*C. flava*	[64]
Pregnane glycosides (nizwaside)	Sap	Anticancer Antidiabetic Antiulcer	*C. flava*	[64, 65]
Pregnane glycosides (carumbelloside-III and dihydro russelioside)	Ethanolic extraction	Antidiabetic Antiobesity Antinociceptive Antiulcer Anti-inflammatory Antiarthritis effects Wound healing activities	*C. pauciflora*	[58, 64]
Pregnane glycoside (russelioside B)	n-Butanol fraction of methanol extract	Antidiabetic Antihyperlipidemic	*C. quadrangular*	[64]
Five pregnane glycosides (caratuberside A-E); pregnant glycoside-russelioside	Chloroform fraction of MeOH extract	Antimalarial Antitrypanosomal Cytotoxic potential	*C. tuberculata*	[64]
Pregnane glycosides (desmiflavasides A and B)	Sap	Antiproliferative Antidiabetic Antiulcer	*C. flava*	[64, 65]
Pregnane glycosides (C15 oxypregnane glycosides (penicillosides A–C))	Chloroform fraction of ethanol extract	—	*C. penicillata*	[64]
Four acylated pregnane glycosides (russeliosides E–H)	Methanolic extraction; chloroform extracts; and n-butanol fraction of ethanol extract	—	*C. penicillata* *C. russeliana*	[64]
Five pregnane derivatives	HPLC-UV LC/MS-TOF	—	*C. fimbriata* *C. attenuata* *C. umbellata*	[66]
Polyoxy pregnane glycoside (retrospinoside 1)	n-butanol fraction of methanol extract; ether extracts	High cytotoxic activity	*C. retrospections*	[64]
Bisdesmosidic C21 pregnane steroidal glycosides (lasianthosides-A and B)	n-Butanol fraction of ethanolic extract; less polar solvent extraction	—	*C. lasiantha*	[64]
Pregnane steroidal glycoside (androstan glycoside)	Ethyl acetate extract	Moderate cytotoxic activity	*C. tuberculata*	[64]
Steroidal glycosides (stalagmoside I–IV)	Butanol fraction	Anti-inflammatory	*C. stalagmifera* *C. indica*	[64]
Steroidal glycosides (Caradalzieloside A-E)	CHCl3/MeOH	—	*C. dalzielii*	[64]
Flavones glycoside (luteolin-4-O-neohesperiodoside)	Methanolic extract; an n-butanol fraction of ethanol extract	—	*C. lasiantha* *C. russeliana*	[64]
Flavones glycoside (megastigmane glycosides)	Methanolic extract	Anti-inflammatory	*C. negevensis*	[64]
Flavone glycosides	Methanolic extract;	Antioxidant	*C. negevensis* *C. attenuate*	[64]
Steroids/triterpenoids; pentacyclic triterpenoid	n-Hexane; butanone, ethylene acetate, and n-butanol; ethanolic extraction; and chloroform extract	Antiapoptotic Neuroprotective Antioxidative Anticancer Anti-inflammatory	*C. attenuate* *C. nilagiriana* *C. russeliana* *C. edulis*	[64, 67–69]
Stigmasterol	Less polar solvent extraction	Anti-inflammatory Antiasthmatic Antioxidative Antiproliferative Neuroprotective	*C. lasiantha* *C. wissmannii*	[64, 70, 71]
Two sterols	Chloroform extract	—	*C. russeliana*	[64]
Flavonoids	Butanone, ethylene acetate, and n-butanol; ethanolic extraction; and chloroform extract	Antioxidant Antitumor Antimutagenic	*C. attenuate* *C. nilagiriana* *C. sinaica* *C. umbellate* *C. tuberculata* *C. edulis*	[64, 67, 72]
Rutin	Ethanolic extraction	Antidiabetic Antibacterial Anti-inflammatory Cardioprotective	*C. nilagiriana* *C. arabica*	[64, 73]
Alkaloids	Ethanolic extraction	Antiadipogenic	*C. nilagiriana* *C. tuberculata* *C. edulis*	[45, 64, 67, 72, 74]
Tannins	Ethanolic extraction	Anti-inflammatory Antioxidant Antidiabetic Anticancer Cardioprotective	*C. nilagiriana* *C. umbellate* *C. edulis*	[50, 64, 67]
Quercetin	—	Secondary metabolite Cardioprotective Neurological protection Antioxidant	*C. arabica*	[64, 74–76]
Polyphenols/phenolic compounds	Ethanolic extraction	Antioxidant	*C. nilagiriana* *C. edulis*	[64, 67]
Saponins	Butanone, ethylene acetate, and n-butanol	Immune system enhancers	*C. attenuate* *C. umbellate* *C. tuberculata* *C. edulis*	[64, 67, 72]

**Table 4:** Different bioactive compounds of *C. fimbriata* against MS.

Target health problem	Bioactive compound	Mechanism of action	Reference
Diabetes mellitus	Pregnane glycoside	The hypoglycemic action is mainly due to the lowering of intestinal glucose absorption or stimulating pancreatic insulin production	[58]
	Quercetin	Stimulate the glucose uptake resulted in the translocation of glucose transporter 4 Reduced the production of sugars by downregulating the key gluconeogenesis enzymes	[82]
	Rutin	Improves insulin secretions Restore glycogen content Inhibit the formation of the advanced glycation end product	[83]
	Saponin	Induces insulin production Oxidative stress amelioration	[83]

Hyperlipidemia	Flavonoids	Exhibit inhibitory effect against pancreatic lipase	[26]
	Quercetin	Able to inhibit lipid peroxidation Decreased the production of reactive oxygen species and act as an anti-inflammatory Inhibit the production of lipoxygenase and cyclooxygenase which are induced by inflammation	[82]

Hypertension	Flavonoids	Able to modulate blood pressure by restoring the endothelial function or by affecting nitric oxide levels	[84]

Obesity	Pregnane glycoside	Aids in fat burning and hunger suppression Works by inhibiting the citrate lyase enzyme and stops the body to produce fat Inhibit malonyl-CoA and block fat synthesis and helps in burning of stored fat resulting in weight loss	[85]
	Quercetin	Decrease the action of an enzyme related to adipogenesis Downregulate the apoptosis while upregulating substrate acetyl-CoA carboxylase	[82]

**Table 5:** Various studies of *C. fimbriata's* therapeutic potential against MS.

Main component	Study subject	Material and method	Result	References
CFE	Overweight adults	Daily supplementation (16 weeks)	A significant effect on body weight maintenance was observed	[20]
CFE	Adults (97)	500 mg for 8 weeks	Significant reduction in stress and anxiety	[28]
CFE	A 14-year-old female (PWS)	CFE supplementation over 12 years	Significant effect against hyperphagia and obesity	[32]
Hydroalcoholic extract of *C. fimbriata*	Animal model (40 rats)	HFD-induced cardiac damage was analyzed	Cardiac protective outcomes were observed	[62]
*C. fimbriata*	Human colon cancer cells	MTT cell viability assay was performed on KB cell lines	Good antiproliferative activity against KB mouth cell line	[88]
Hydroalcoholic extract of *C. fimbriata*	Animal model (Wister rats)	Oxidative stress markers GSH, LO, PO, SDH, and AR were examined	Reduced oxidative and pancreatic damage caused by HFD	[89]
Hydroalcoholic extract of *C. fimbriata*	Animal model (HFD diabetic rats)	Carbohydrate metabolism was analyzed in rats with HFD	Significantly restore the levels of glycogen in the liver and muscles	[18]
Ethanolic leaf extract of *C. fimbriata*	Human colon cancer cell	Antiproliferative effects were evaluated using MTT assays	Reduced cell proliferation by inducing cytotoxicity of COLO 320 cells	[88]
Commercially available CFE	Animal model (female rats)	Modulation of brain neuropeptides NPY and ORX	Significant reduction in weight gain	[55]
Ethanolic leaf extract of *C. fimbriata*	Vitro approach	Alpha-amylase and alpha-glucosidase inhibitory assay with acarbose as control	Potent antihyperglycemic activity	[23]
Hydroalcoholic extract of *C. fimbriata*	Animal model (male Wister rats)	Renal functional and oxidative stress markers were checked	Effectively alleviated the HFD-induced renal damage	[35]
CFE and metformin	Wister rats	Lipid profile was analyzed	Significant reduction in lipid profile	[39]
CFE	Male Wister rats	Oxidative stress markers were checked	Significant protection against HF diet	[34]
CFE	Animal and human	Snord116 deletion	Significant alteration in appetite	[90]
CFE	PWS children and adolescents	Appetite behavior was recorded	Significant reduction in hyperphagia	[30]
CFE	Overweight and obese individuals	Anthropometry, appetite, and biochemical investigation done	No significant changes in the biochemical and clinical parameters	[91]
CFE	Animal model	Glucose, leptin, and triglycerides were measured	Significant reduction in insulin resistance and oxidative stress	[14]
CFE	Animal model	Metabolic parameters were assessed	Significant reduction in food intake and blood pressure	[92]
CFE	Animal model	Hepatotoxic, diabetic, and renal toxicities were analyzed	Significant reduction in diabetes	[87]
CFE	Animal model	Renal and liver function tests were measured	Significant reduction in body weight and lipid profile	[93]
CFE	Animal model	Serum lipid profile and blood glucose were measured	Significant alteration in lipid profile and body weight	[94]
CFE	Overweight and obese individuals	500 mg capsules twice for 12 weeks	CFE showed a reduction in BMI, weight gain, hip circumference, and systolic blood pressure	[95]
